# Homeostatic control of *Drosophila* neuromuscular junction function

**DOI:** 10.1002/syn.22133

**Published:** 2019-10-04

**Authors:** C. Andrew Frank, Thomas D. James, Martin Müller

**Affiliations:** ^1^ Department of Anatomy and Cell Biology University of Iowa Carver College of Medicine Iowa City Iowa USA; ^2^ Interdisciplinary Programs in Neuroscience, Genetics, and Molecular Medicine University of Iowa Iowa City Iowa USA; ^3^ Interdisciplinary Graduate Program in Neuroscience University of Iowa Iowa City Iowa USA; ^4^ Institute of Molecular Life Sciences University of Zurich Zurich Switzerland; ^5^ Neuroscience Center Zurich Zurich Switzerland; ^6^ Present address: Department of Neuroscience and Pharmacology University of Iowa Carver College of Medicine Iowa City Iowa USA

**Keywords:** homeostatic plasticity, neurotransmitter release, presynaptic mechanisms, synaptic plasticity, transsynaptic signalling

## Abstract

The ability to adapt to changing internal and external conditions is a key feature of biological systems. Homeostasis refers to a regulatory process that stabilizes dynamic systems to counteract perturbations. In the nervous system, homeostatic mechanisms control neuronal excitability, neurotransmitter release, neurotransmitter receptors, and neural circuit function. The neuromuscular junction (NMJ) of *Drosophila melanogaster* has provided a wealth of molecular information about how synapses implement homeostatic forms of synaptic plasticity, with a focus on the transsynaptic, homeostatic modulation of neurotransmitter release. This review examines some of the recent findings from the *Drosophila* NMJ and highlights questions the field will ponder in coming years.

## Graphical abstract

At the larval *Drosophila* neuromuscular junction (NMJ), neurotransmitter receptor (blue) perturbation in the muscle cell (gray cylinder) enhances neurotransmitter release (green, “Presynaptic homeostatic potentiation”, PHP) from the motor neuron. This review summarizes recent updates on this evolutionarily conserved form of transsynaptic plasticity.

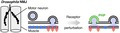

## 1 INTRODUCTION

Most biological systems rely on homeostatic mechanisms to maintain robust function when faced with perturbations. For daily living, key physiological parameters, such as body temperature or water/electrolyte balance, are under homeostatic control. In the nervous system, metazoans have evolved homeostatic mechanisms to actively stabilize neuronal excitability, chemical synaptic transmission, and neural circuit function (Delvendahl & Müller, 2019; Marder & Goaillard, 2006; Pozo & Goda, 2010; Turrigiano, 2008). A marvelous diversity of homeostatic processes controlling neural function has been identified: Homeostatic mechanisms compensate for activity manipulations of single neurons (Burrone, O'Byrne, & Murthy, 2002; Murthy, Schikorski, Stevens, & Zhu, 2001) or neural networks in vitro (Hartman, Pal, Burrone, & Murthy, 2006; O'Brien et al., 1998; Turrigiano, Leslie, Desai, Rutherford, & Nelson, 1998) and in vivo (Desai, Cudmore, Nelson, & Turrigiano, 2002; Maffei & Turrigiano, 2008). Homeostatic signaling controls neural activity on various space scales, ranging from individual synaptic spines (Béïque, Na, Kuhl, Worley, & Huganir, 2011), dendritic branches (Branco, Staras, Darcy, & Goda, 2008), to entire neurons (Turrigiano et al., 1998), or networks of neurons (Marder & Goaillard, 2006). In most cases, homeostatic compensation is studied after prolonged neural activity perturbations for hours to days (Pozo & Goda, 2010), but there is also evidence for more rapid forms of homeostatic signaling in the peripheral nervous system (Frank, Kennedy, Goold, Marek, & Davis, 2006; Wang, Pinter, & Rich, 2016).

At the level of synapses, there is evidence for homeostatic regulation of neurotransmitter release (Cull‐Candy, Miledi, Trautmann, & Uchitel, 1980; Davis & Goodman, 1998; Petersen, Fetter, Noordermeer, Goodman, & DiAntonio, 1997) and neurotransmitter receptor abundance/function (Turrigiano et al., 1998; Wierenga, Ibata, & Turrigiano, 2005). Homeostatic regulation of neurotransmitter release, often called presynaptic homeostatic plasticity, has been described for neuromuscular synapses in different species (Cull‐Candy et al., 1980; Petersen et al., 1997; Plomp, van Kempen, & Molenaar, 1992) and several mammalian central nervous system (CNS) synapses (Burrone et al., 2002; Zhao, Dreosti, & Lagnado, 2011). Presynaptic homeostatic plasticity involves modulation of presynaptic Ca^2+^ influx (Frank et al., 2006; Glebov et al., 2017; Jeans, van Heusden, Al‐Mubarak, Padamsey, & Emptage, 2017; Müller & Davis, 2012; Zhao et al., 2011) and the size of the readily releasable pool (RRP) (Müller, Liu, Sigrist, & Davis, 2012; Wang, Pinter, et al., 2016; Weyhersmüller, Hallermann, Wagner, & Eilers, 2011) or the recycling pool of synaptic vesicles (Jeans et al., 2017; Kim & Ryan, 2010). Thus, there likely exist ancient presynaptic homeostatic plasticity mechanisms.

**Figure 1:**
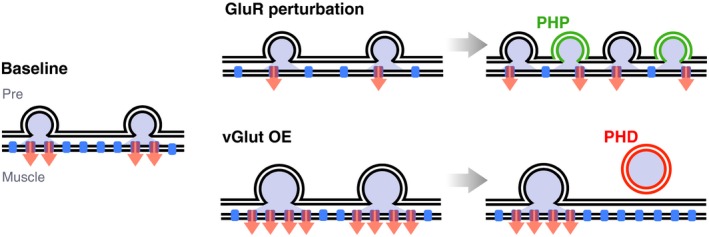
Presynaptic homeostatic plasticity. *Top* At the *Drosophila* NMJ, pharmacological or genetic glutamate receptor (GluR, blue) perturbation (illustrated as decreased GluR number) induces presynaptic homeostatic potentiation (PHP) of neurotransmitter release. PHP maintains AP‐induced postsynaptic muscle excitation around baseline levels (red arrows). *Bottom* Presynaptic overexpression (OE) of the vesicular glutamate transporter vGlut elevates neurotransmitter content per synaptic vesicle (increased vesicle diameter) and induces presynaptic homeostatic depression (PHD) of neurotransmitter release, thereby stabilizing AP‐evoked muscle depolarization (red arrows)

The identification of the molecular pathways underlying homeostatic plasticity is especially important because of emerging links between homeostatic maintenance of neural function and several neurological conditions, such as epilepsy, schizophrenia (Bliss, Collingridge, & Morris, 2014; Wondolowski & Dickman, 2013), or autism spectrum disorders (Mullins, Fishell, & Tsien, 2016). However, little is known about the molecular mechanisms underlying presynaptic homeostatic plasticity in the mammalian CNS. Instead, the signaling systems controlling presynaptic homeostatic plasticity have been most extensively studied at the larval NMJ of *Drosophila melanogaster* (Delvendahl & Müller, 2019). In this preparation, genetic or pharmacological perturbation of glutamatergic neurotransmitter receptors results in an increase in neurotransmitter release. Remarkably, the increase in neurotransmitter release precisely scales with the degree of receptor impairment, thereby maintaining action potential (AP)‐induced postsynaptic excitation at control levels—that is, in the absence of receptor perturbation (Frank et al., 2006; Petersen et al., 1997; Figure 1). Intriguingly, this homeostatic upregulation of release can occur within minutes after receptor perturbation (Frank et al., 2006).

The possibility of acute pharmacological induction and rapid expression of presynaptic homeostatic plasticity in the genetic model organism *Drosophila* (Frank et al., 2006) opened the door for genetic screens that are based on electrophysiological analysis of synaptic transmission (Brusich, Spring, & Frank, 2015; Dickman & Davis, 2009; Hauswirth et al., 2018; Kikuma et al., 2019; Müller, Pym, Tong, & Davis, 2011). At this point, we are able to take a retrospective view of these screens and the resulting characterized molecules. We note that around 2,000 genetic lines have been examined, and more than three dozen genetic perturbations with impaired presynaptic homeostatic plasticity have been uncovered. When scrutinized for further study, the large majority of the identified genes has been verified using multiple genetic alleles or loss‐of‐function conditions. Many of the positives that emerged from these genetic screens point to discrete presynaptic processes that regulate neurotransmitter release. Less is known about the postsynaptic processes that drive homeostatic signaling, but new clues are emerging with regularity.

Here we review recent findings in the field of presynaptic homeostatic plasticity at the *Drosophila* NMJ. Given the progress in the field and the pace of discovery, we consider an update to be timely. This updated summary should be viewed as a companion to prior reviews (Davis & Müller, 2015; Delvendahl & Müller, 2019; Wondolowski & Dickman, 2013; Frank, 2014a, 2014b). Parallel work on homeostatic plasticity continues apace at the mammalian NMJ (Homan & Meriney, 2018) and mammalian CNS preparations (Li, Park, Zhong, & Chen, 2019; Wefelmeyer, Puhl, & Burrone, 2016).

## 2 NEW PHENOMENOLOGY

### 2.1 Reversibility and temperature sensitivity

Reversibility is a hallmark of homeostatic systems. For synaptic homeostasis, the idea of reversibility is straightforward: If synaptic transmission is under homeostatic control, and if a specific perturbation of synaptic function initiates a homeostatic signal, then the effects of that signal should be reversed once the perturbation is removed. The fact that both presynaptic homeostatic potentiation (PHP) and presynaptic homeostatic depression (PHD, Figure 1; Daniels et al., 2004) occur at the *Drosophila* NMJ means that this synapse has the capacity to bidirectionally regulate neurotransmitter output. A formal demonstration of homeostatic reversibility at the *Drosophila* NMJ has not been straightforward. Since synaptic activity perturbations like glutamate receptor subunit gene deletion (Petersen et al., 1997) or knockdown (Brusich et al., 2015) persist throughout development, they cannot be simply removed.

Conditional expression of a dominant‐negative glutamate receptor subunit transgene (*UAS‐GluRIIA^M/R^*) circumvented this problem. Continuous postsynaptic expression of *UAS‐GluRIIA^M/R^* reduces quantal size and induces PHP (DiAntonio, Petersen, Heckmann, & Goodman, 1999). A recent study (Yeates, Zwiefelhofer, & Frank, 2017) combined the temperature‐sensitive *GAL4/GAL80^TS^* expression system (McGuire, Le, Osborn, Matsumoto, & Davis, 2003) with *UAS‐GluRIIA^M/R^* expression. It was found that expression of the dominant‐negative glutamate receptor subunit at the beginning of development initiates PHP, and that PHP is turned off after turning off the expression of the dominant‐negative glutamate receptor subunit, over a timescale of two to three days (Yeates et al., 2017). These data demonstrate that PHP is reversible at the *Drosophila* NMJ. An additional and unexpected finding was that if the ambient temperature is too high, the long‐term expression of PHP fails, likely due to aberrant synapse development (Yeates et al., 2017). The temperature sensitive nature of this system was also previously suggested by a blunted NMJ growth phenotype at high rearing temperature for *GluRIIA* loss‐of‐function mutants (Sigrist, Reiff, Thiel, Steinert, & Schuster, 2003). Collectively, the data suggest that there are limits to the homeostatic capacity of the NMJ; if the synapse is facing high temperature and concomitant receptor subunit loss, then the homeostatic mechanisms in place to maintain postsynaptic excitation over chronic developmental time periods are no longer able to fully compensate.

The reversibility time course of two to three days after the genetic manipulations described above is limited by the half‐life of glutamate receptors (Yeates et al., 2017). It would be desirable to test if PHP were reversible on shorter time scales. An intuitive way to test this would be pharmacology. PHP can be induced on a timescale of 5–10 min by application of the glutamate receptor antagonist Philanthotoxin‐433 (PhTx), which causes noncompetitive, use‐dependent inhibition of glutamate receptors at the *Drosophila* NMJ (Frank et al., 2006). However, since a significant fraction of PhTx irreversibly blocks glutamate receptors at the *Drosophila* NMJ (Frank et al., 2006), PhTx cannot be used to study the reversibility of PHP.

This technical challenge for the *Drosophila* NMJ was recently solved at the vertebrate NMJ. Loss of human nicotinic acetylcholine receptors during the autoimmune disease myasthenia gravis (Cull‐Candy et al., 1980) or pharmacological inhibition of rodent nicotinic acetylcholine receptors (Plomp et al., 1992) results in PHP, similar to the *Drosophila* NMJ. By reversibly applying the drug D‐Tubocurarine (D‐TC) to dissected mouse tibialis anterior NMJs, researchers showed that the timescales of PHP induction, expression, and reversal are fast, all occurring within minutes of D‐TC exposure and washout (Wang, Pinter, et al., 2016; Wang, McIntosh, & Rich, 2018). It remains unknown if PHP reverses on similar time scales at the *Drosophila* NMJ. Yet, taken together, these studies suggest that PHP is reversible at the mouse and *Drosophila* NMJ, fulfilling a key criterion of homeostatic systems. It will be exciting to test links between the molecular mechanisms underlying rapid PHP induction, expression, and reversal.

### 2.2 Target and input specificity

**Figure 2:**
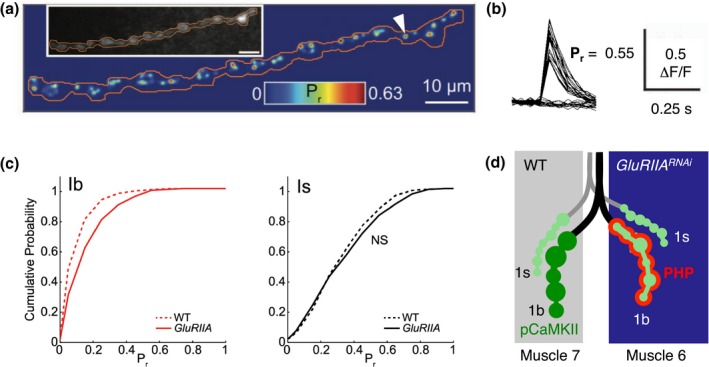
Input and target specificity of PHP. (a) Cumulative AP‐evoked quantal release location heat map derived from postsynaptic Ca^2+^ imaging at the *Drosophila* NMJ (SynapGCaMP6f; 200 trials at 0.1 Hz). Inset shows baseline SynapGCaMP6f fluorescence. Local release probability (P_r_ = number of responses/number of trials at individual sites) is represented as a color scale. Reprinted and adapted from (Newman et al., 2017) with permission from Elsevier. (b) Ca^2+^ imaging traces (ΔF/F) for the synapse indicated with the arrowhead in (a) during 40 trials. Reprinted and adapted from (Newman et al., 2017) with permission from Elsevier. (c) Cumulative probability for pooled evoked single synapse P_r_ at wild‐type (WT) and *GluRIIA^SP16^* 1b NMJs (left) and 1s NMJs (right). Note the increased P_r_ at type 1b boutons of *GluRIIA* mutants. Reprinted and adapted from (Newman et al., 2017) with permission from Elsevier. (d) Cartoon illustrating PHP input and target specificity. At the *Drosophila* NMJ, two motor neurons (“type 1s” and “type 1b” synapses) innervate two muscle cells (“Muscle 6” and “Muscle 7”). PHP (red) is predominantly expressed at type 1b motor neuron boutons contacting the muscle cell with perturbed glutamate receptor function (“*GluRIIA^RNAi^*”; *G‐14‐Gal4 > UAS‐GluRIIA^RNAi^*, (Li, Goel, Chen, et al., 2018). This is correlated with reduced phosphorylated CaMKII levels (“pCaMKII,” green; light green indicates reduced pCaMKII levels)

A long‐standing question in the field of homeostatic plasticity has been how homeostatic signaling controls synaptic transmission on a spatial scale. Do homeostatic mechanisms act “locally” at the level of individual synaptic connections, or “globally” over a range of synapses and circuit hierarchies? At the larval *Drosophila* NMJ, most muscle cells receive convergent afferent input from two motor neuron types. These two types of motor neurons either form “type 1b” or “type 1s” boutons with a low or high baseline release probability (P_r_), respectively (Figure 2d; Kurdyak, Atwood, Stewart, & Wu, 1994). Hence, this system allows investigating how glutamate receptor perturbation in the postsynaptic muscle cell affects release from two distinct inputs. Newman and colleagues (2017) employed postsynaptic Ca^2+^ imaging at the *Drosophila* NMJ to assess presynaptic P_r_ of synapses formed by two motor neurons that provide convergent input to the same muscle cell (Figure 2a–d; Newman et al., 2017). They uncovered that genetic glutamate receptor perturbation augmented P_r_ from the motor neuron with low baseline P_r_ (1b boutons; Figure 2c,d). By contrast, release from the neuron with high baseline P_r_ (1s boutons) was largely unchanged after glutamate receptor inhibition (Figure 2c,d). These data indicate that presynaptic homeostatic plasticity is “input specific” at the *Drosophila* NMJ, at least after genetic receptor perturbation (Figure 2d). The study also revealed a decrease in postsynaptic phosphorylated CaMKII levels (pCaMKII) upon genetic glutamate receptor impairment, which occurred opposite to synapses made by 1b boutons with increased P_r_ (Newman et al., 2017). These results suggest an input‐specific, negative relationship between the degree of homeostatic P_r_ potentiation and postsynaptic CaMKII phosphorylation upon genetic glutamate receptor perturbation. It remains to be determined if the homeostatic increase in release from the motor neuron with low baseline P_r_ alone is sufficient to maintain AP‐induced postsynaptic potential changes at control levels.

The *Drosophila* NMJ also permits testing if compensatory release modulation is “target‐specific,” because most motor neurons innervate more than one muscle cell. Davis and Goodman (1998) biased innervation of a motor neuron contacting two postsynaptic muscles toward one muscle by overexpressing Fascilin II in the respective muscle cell (Davis & Goodman, 1998). Remarkably, the motor neuron had reduced P_r_ onto the hyperinnervated muscle, while the hypoinnervated muscle showed increased quantal size. These results imply that homeostatic modulations are target specific. In the case of hypoinnervation and increased quantal size, the phenomenon is somewhat reminiscent of homeostatic modulations of receptor abundance reported for mammalian synaptic preparations (Turrigiano et al., 1998). Indeed, a recent paper at the *Drosophila* NMJ demonstrated that the increased quantal size of hypoinnervated muscle cells is due to increased glutamate receptor abundance (Goel & Dickman, 2018).

Another recent study extended these concepts by investigating the “target specificity” of PHP upon glutamate receptor inhibition (Li, Goel, Chen, et al., 2018). The authors downregulated the GluRIIA subunit by RNA interference in only one of two muscle cells innervated by the same motor neuron (Figure 2d). It was found that release is predominantly augmented at active zones of motor neuron branches contacting the muscle with impaired glutamate receptor function (Figure 2d). This implies that a given presynaptic motor neuron can differentially regulate release depending on the glutamate receptor function of the postsynaptic partner cell. The same study provided evidence for target‐specific homeostatic modulation of phosphorylated CaMKII levels, RRP size, and functional release sites. The results of this investigation suggest that PHP induction and expression mechanisms are locally transmitted and restricted to specific branches of the presynaptic motor neuron and the postsynaptic muscle cell with reduced glutamate receptor activity. Together, the experimental evidence argues against “global,” cell‐wide PHP signaling at the *Drosophila* NMJ. It will be interesting to assess if local PHP signaling occurs even at smaller space scales, possibly at the level of individual active zones and postsynaptic densities.

### 2.3 PHD versus PHP

Most work on presynaptic homeostatic plasticity has focused on the mechanisms of homeostatic potentiation of release upon neural activity perturbation (PHP; Figure 1). Yet, there is also evidence for presynaptic homeostatic depression of release (PHD; Figure 1). Overexpression of the vesicular glutamate transporter vGlut in *Drosophila* motor neurons causes larger glutamatergic vesicles (Daniels et al., 2004). As a result, one observes increased quantal size and decreased quantal content, thereby precisely maintaining AP‐evoked postsynaptic potentials at baseline levels (Figure 1). It had remained elusive if opposing mechanisms drive PHP and PHD. On the one hand, the two processes result in the exact opposite outcomes with regard to presynaptic release. On the other hand, there are distinct differences between the induction of PHP and PHD. While PHP can be induced by postsynaptic receptor impairment (see Frank, 2014a, for a summary), there is so far no evidence that PHD compensates for postsynaptic perturbations that increase quantal size at the *Drosophila* NMJ (Davis, DiAntonio, Petersen, & Goodman, 1998; DiAntonio et al., 1999; Petersen et al., 1997). This brings up the question if PHD is achieved through retrograde signaling mechanisms.

A recent study demonstrated that the vGlut overexpression‐induced decrease in release during PHD correlates with reduced levels of a transgenically expressed GFP‐tagged Ca_V_2 Ca^2+^ channel subunit (Cacophony‐GFP) and decreased AP‐induced presynaptic Ca^2+^ influx (Gaviño, Ford, Archila, & Davis, 2015). As PHP requires enhanced presynaptic Ca^2+^ influx (Frank et al., 2006; Müller & Davis, 2012) that is correlated with increased levels of voltage‐gated Ca^2+^ channels (Gratz et al., 2019; Li, Goel, Wondolowski, Paluch, & Dickman, 2018), these data suggest that PHD could be implemented in an opposite type of mechanism as PHP. However, the same study (Gaviño et al., 2015), as well as another recent study (Li, Goel, Wondolowski, et al., 2018), uncovered that genes that are required for PHP are dispensable for PHD, implying—at least in part—different molecular pathways. Moreover, both studies revealed that glutamate receptor perturbation still results in enhanced release at vGlut‐overexpressing synapses, suggesting that these two forms of synaptic plasticity act independently to bidirectionally modulate presynaptic release in a homeostatic fashion. Finally, unlike PHP, PHD does not seem to be an input specific process (Li, Goel, Wondolowski, et al., 2018).

New work has reported that endogenous synaptic protein levels of the active zone protein Bruchpilot (Brp, an ELKS/CAST homolog, (Kittel et al., 2006)) and Ca_V_2/Cacophony do not change upon PHD induction (Gratz et al., 2019). These data are consistent with a model in which the deceased release associated with PHD is executed—at least in part—through functional modulations of existing active zone components. Are those modulations executed through muscle‐to‐nerve retrograde signaling? Work from Li and colleagues posits that an autocrine glutamate homeostat might be responsible for effecting PHD at the NMJ (Li, Goel, Wondolowski, et al., 2018). This model awaits affirmative data—that is, a mechanism that signals excess cleft glutamate to the motor neuron to dampen presynaptic release.

There are relevant parallels to vertebrate models. At mouse hippocampal synapses, prolonged elevation of neural activity upon Gabazine treatment induces a homeostatic decrease in presynaptic Ca^2+^ influx and release (Jeans et al., 2017; Zhao et al., 2011), which is mediated by the activity of Ca_V_2.1 (P/Q)‐type Ca^2+^ channels (Jeans et al., 2017). There is also evidence for a homeostatic reduction of neurotransmitter release after prolonged depolarization at hippocampal synapses (Moulder, Jiang, Taylor, Olney, & Mennerick, 2006). This reduction depends on the ubiquitin–proteasome system (UPS) and is correlated with decreased protein levels of Munc13‐1 and Rim1 (Jiang et al., 2010). Moreover, effective recovery from homeostatic reduction of release depends on adenylyl cyclase activity (Conti et al., 2009). Thus, PHD can be induced by several activity perturbations and may involve different molecular mechanisms. Future work should address if and how these different forms of PHD are related to one another.

## 3 NEW GENES AND MECHANISMS

### 3.1 Presynaptic mechanisms

#### 3.1.1 Physiology and genes

**Figure 3:**
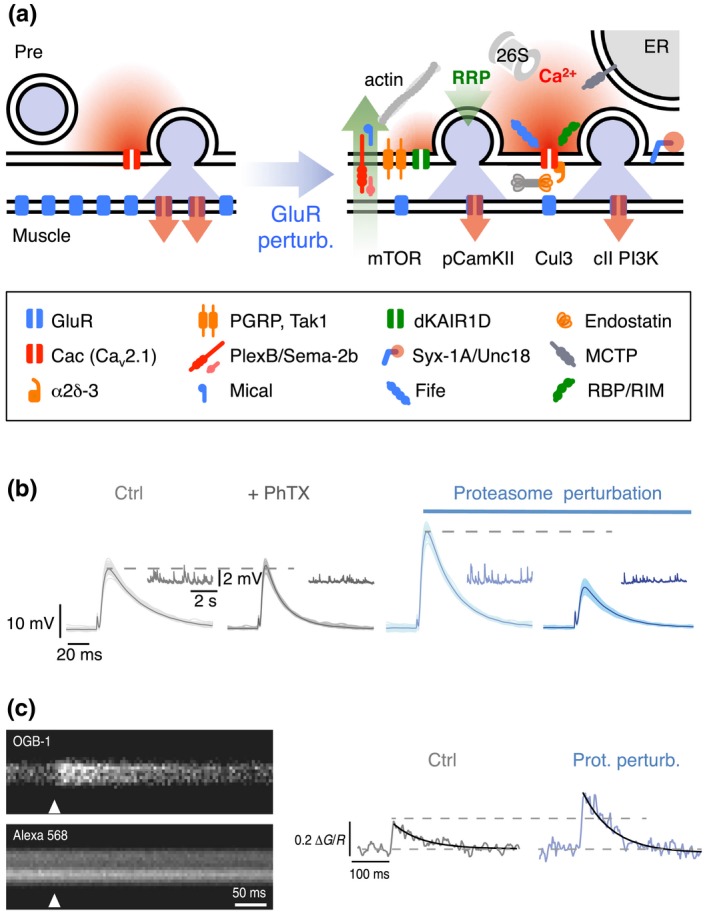
Molecular mechanisms underlying PHP. (a) Cartoon of a synapse under control conditions (left) and after GluR perturbation (right). Glutamate receptor perturbation enhances presynaptic Ca^2+^ influx (red) (Frank et al., 2006; Müller & Davis, 2012) and RRP size (green) (Weyhersmüller et al., 2011). *Ca_v_2.1* (*cacophony*, *cac*; Frank et al., 2006; Müller & Davis, 2012), *α2δ‐3* (Wang, Jones, et al., 2016), *endostatin*/*multiplexin* (Wang et al., 2014), and rim‐binding protein (*rbp*) (Müller et al., 2015) have been implicated in homeostatic regulation of presynaptic Ca^2+^ influx. The following genes have been implicated in RRP size regulation under baseline conditions and/or during PHP: The presynaptic proteasome (“26S”) (Wentzel et al., 2018), *fife* (Bruckner et al., 2017), *mctp* (Genç et al., 2017), *mical* (Orr et al., 2017), *pgrp, tak1* (Harris et al., 2015, 2018), *dKaiR1D* (Kiragasi et al., 2017), *α2δ‐3* (Wang, Jones, et al., 2016), *plexB/sema2b* (Orr et al., 2017), *syntaxin-1A* (*syx-1A*), *unc18* (*rop*) (Ortega et al., 2018), *rbp* (Müller et al., 2015), and *rim* (Müller et al., 2012). Retrograde PHP signaling involves *multiplexin/endostatin* (Wang et al., 2014) and Sema‐2B/Plexin B (Orr et al., 2017). PHP requires postsynaptic mTOR signaling (Goel et al., 2017; Penney et al., 2012), class II PI3 kinase function (Hauswirth et al., 2018), and reduced pCaMKII levels (Goel et al., 2017; Li, Goel, Chen, et al., 2018; Newman et al., 2017). Note that the cartoon only summarizes recent genes implicated in PHP. More molecular PHP mechanisms are reviewed in (Davis & Müller, 2015; Delvendahl & Müller, 2019; Wondolowski & Dickman, 2013; Frank, 2014a). (b) At wild‐type NMJs (gray), application of the glutamate receptor antagonist philanthotoxin‐433 (“PhTX”) decreases miniature EPSP amplitudes (inset) and enhances presynaptic release, thereby maintaining AP‐evoked EPSP amplitudes at control levels. Acute or sustained proteasome perturbation (blue) enhances presynaptic release in the absence of glutamate receptor inhibition and blocks PHP. Reprinted and adapted from (Wentzel et al., 2018) with permission under a Creative Commons Attribution 4.0 License (https://creativecommons.org/licenses/by/4.0/). (c) Presynaptic Ca^2+^ imaging (motor neuron boutons were loaded with the nonmembrane permeable Ca^2+^ indicator Oregon‐Green‐BAPTA‐1, “OGB‐1,” and the reference dye Alexa 568) revealed that presynaptic proteasome perturbation (*elav^c155^‐Gal4 > UAS‐DTS*) results in increased amplitudes of presynaptic Ca^2+^ transients upon single AP stimulation. These data suggest that presynaptic proteasomal degradation has the capacity to regulate Ca^2+^ influx. Reprinted and adapted from (Wentzel et al., 2018) with permission under a Creative Commons Attribution 4.0 License (https://creativecommons.org/licenses/by/4.0/)

Most work on presynaptic homeostatic plasticity at the *Drosophila* NMJ has focused on the presynaptic mechanisms underlying PHP. Two major presynaptic parameters are modulated during PHP—RRP size and P_r_ (Davis & Müller, 2015). There is evidence that homeostatic P_r_ potentiation is driven by enhanced presynaptic Ca^2+^ influx (Müller & Davis, 2012; Figure 3a). Several genes, which have been discovered by genetic screens, have been linked to homeostatic regulation of RRP size and/or P_r_ (for a recent review see Delvendahl & Müller, 2019). Two studies identified the first genes that are required for both, the homeostatic control of RRP size and presynaptic Ca^2+^ influx. These genes encode RIM‐binding protein (RBP; Müller, Genç, & Davis, 2015) and the auxiliary voltage‐gated Ca^2+^ channel subunit α2δ‐3 (Wang, Jones, Whippen, & Davis, 2016), two proteins that biochemically interact with presynaptic voltage‐gated Ca^2+^ channels. There is also evidence that RIM, which also binds to the C‐terminus of voltage‐gated Ca^2+^ channels, participates in homeostatic regulation of RRP size, but not presynaptic Ca^2+^ influx (Müller et al., 2012). This suggests that the homeostatic modulations of RRP size and presynaptic Ca^2+^ influx are genetically separable.

A major factor determining P_r_ is the relative “coupling distance” between voltage‐gated Ca^2+^ channels and the vesicular Ca^2+^ sensor for synaptic vesicle fusion. The slow Ca^2+^ chelator EGTA can be used to assess “Ca^2+^ influx‐release coupling,” because it predominantly interferes with the release of synaptic vesicles that are “loosely‐coupled” to Ca^2+^ influx. Several studies revealed that PHP is disrupted after loss of molecules that confer tight coupling between presynaptic Ca^2+^ influx and release under baseline conditions, such as α*2*δ*‐3* (T. Wang, Jones, et al., 2016)*, mctp* (Genç et al., 2017)*, rbp* (Müller et al., 2015), and *rim* (Müller et al., 2012). One of the recently discovered genes promoting tight coupling and PHP is *fife*, a *Drosophila* Piccolo‐RIM homolog (Bruckner et al., 2017; Figure 3a). In addition to tightly‐coupled synaptic vesicles, recent experimental evidence suggests a role for “loosely‐coupled” vesicles in PHP (Wentzel, Delvendahl, Sydlik, Georgiev, & Müller, 2018). Specifically, it was found that release at NMJs undergoing PHP is more sensitive to EGTA‐AM, and that EGTA‐sensitive vesicles are required for PHP (Wentzel et al., 2018) (but see Genç et al., 2017). This implies that loosely coupled vesicles have to be recruited in addition to tightly coupled vesicles to potentiate release during PHP (see also paragraph “Proteostasis”).

Among the more recently identified genes that are required for homeostatic release modulation is *pgrp*, a gene encoding an evolutionarily conserved innate immune receptor (Harris et al., 2015). The authors demonstrate that presynaptic PGRP is required for homeostatic RRP size expansion (Figure 3a). Moreover, several molecules that act downstream of PGRP were implicated in PHP (Harris, Fetter, Brasier, Tong, & Davis, 2018). It was found that *tak1* (*map3K*) selectively controls the rapid expression of PHP. Together, these findings suggest links between innate immune and PHP signaling. Another gene that has been recently linked to PHP is *mctp* (Multiple C2 Domain Protein with Two Transmembrane Region, Figure 3a; Genç et al., 2017). MCTP was shown to localize to the presynaptic ER, and to regulate homeostatic potentiation of release downstream of presynaptic Ca^2+^ influx (Genç et al., 2017; Figure 3a). The study also demonstrates that MCTP’s C2‐Ca^2+^‐binding domains are required for PHP. Together with the localization of this Ca^2+^ sensor, these results imply a role for ER‐related Ca^2+^ signaling in PHP. In addition, a recent investigation revealed that the concerted action of Unc18, Syntaxin1A and RIM maintain a constant ratio between primed to super‐primed synaptic vesicles during PHP (Ortega, Genç, & Davis, 2018; Figure 3a). Another study implicated an uncharacterized presynaptic glutamate receptor in PHP (Kiragasi, Wondolowski, Li, & Dickman, 2017). Presynaptic expression of this kainate receptor (dKaiR1D) was found to be required for rapid and sustained PHP expression, but not for the acute induction of PHP (Figure 3a). Notably, dKaiR1D localizes to presynaptic active zones, where it was shown to conduct Ca^2+^ (Kiragasi et al., 2017), indicating that autocrine presynaptic Ca^2+^ signaling through this glutamate receptor may participate in PHP. Intriguingly, both, *dKaiR1D* and *mctp* promote PHP at low extracellular Ca^2+^ levels. This indicates that different molecules may control PHP at different Ca^2+^ concentrations. Thus, a number of new genes have been identified to be required for homeostatic regulation of release, and some of these genes have been linked to specific presynaptic mechanisms.

#### 3.1.2 Proteostasis

Despite progress in discovering new genes that are required for PHP, comparably little is known about how the proteins encoded by the identified genes are regulated during PHP. A recent study tested if synaptic proteostasis plays a role in PHP. Wentzel and colleagues (2018) demonstrated that presynaptic protein degradation is needed for rapid and long‐term PHP expression. It was found that synaptic proteasome inhibition increases neurotransmitter release (Figure 3b), presynaptic Ca^2+^ influx (Figure 3c) and RRP size, which occludes release potentiation during PHP (Figure 3b). Moreover, it was shown that the vesicles that are recruited upon proteasome perturbation and PHP are more EGTA sensitive, implying looser Ca^2+^ influx‐release coupling (Wentzel et al., 2018) (see above). Interestingly, homeostatic recruitment of these loosely coupled vesicles requires the schizophrenia susceptibility gene *dysbindin* (*dysb*), a gene previously identified to be required for PHP by a genetic screen (Dickman & Davis, 2009). Future work should address how proteasomal degradation is involved in homeostatic regulation of neurotransmitter release.

#### 3.1.3 Active zone structure

**Figure 4:**
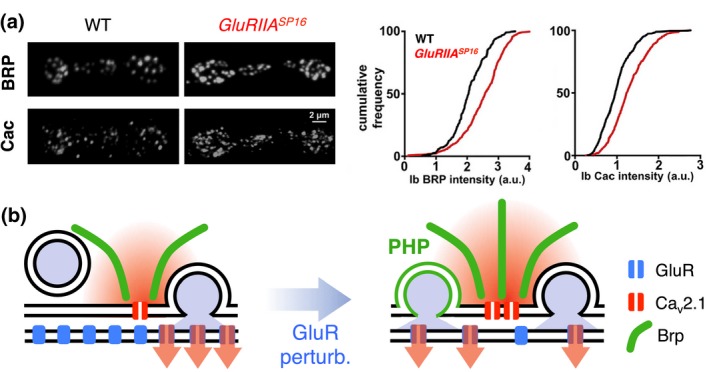
Structural changes during PHP. (a) Confocal images of immunostainings against the presynaptic protein Bruchpilot (BRP) and Cacophony (Cac) of representative NMJs transgenically overexpressing Cac‐GFP (*elav^c155^‐Gal4 > UAS‐cac‐GFP*) in wild type (WT) and *GluRIIA^SP16^* mutants (*GluRIIA^SP16^*). Note the increased BRP and Cacophony fluorescence intensity in *GluRIIA^SP16^* mutants (red data in cumulative frequency plots). Reprinted and adapted from (Li, Goel, Wondolowski, et al., 2018) with permission from Elsevier. (b) Cartoon summarizing structural changes during PHP. GluR (blue) perturbation increases Ca^2+^ channel levels (red) and Brp abundance (green). Further structural changes during PHP are summarized in the section “*Active zone structure*”

How do the physiological changes during PHP manifest on the structural level? Using STED and confocal microscopy, Weyhersmüller and colleagues (Weyhersmüller et al., 2011) provided evidence that the abundance of the presynaptic active zone (AZ) protein bruchpilot (Brp) is slightly increased after acute or chronic glutamate receptor perturbation (Figure 4a,b). Several labs confirmed increased Brp abundance during rapid or sustained PHP expression (Böhme et al., 2019; Goel, Li, & Dickman, 2017; Gratz et al., 2019). Intriguingly, acute and prolonged glutamate receptor perturbation also increases the fluorescence intensity of fluorescently‐tagged presynaptic voltage‐gated Ca^2+^ channels (Figure 4; Gratz et al., 2019; Li, Goel, Wondolowski, et al., 2018). Moreover, the fluorescence intensity of antibody stainings of several other presynaptic proteins—RBP, Unc13A, and Syntaxin‐1A (Syx‐1A)— increased after acute glutamate receptor inhibition (Böhme et al., 2019). This implies that the abundance of these synaptic proteins is modulated on a minutes‐long time scale, depending on the perturbation of glutamate receptors. Rapid remodeling of the synaptic abundance of these proteins was blocked at synapses lacking Brp, RBP, Unc13A, App‐like interacting protein‐1 (aplip‐1), a selective RBP transport‐adaptor, as well as serine–arginine (SR) protein kinase at location 79D (Srpk79D29) Srpk79D (Böhme et al., 2019). Intriguingly, except for *rbp* and *unc13A*, loss‐of‐function mutations in these genes did not impair the rapid expression of PHP, suggesting partial separation between functional and structural changes during PHP expression. Sustained glutamate receptor inhibition in the *GluRIIA^SP16^* mutant background increased the fluorescence intensity of antibodies targeting Brp, RBP, Unc13A, Syx‐1A, and Unc18 (Böhme et al., 2019) or GFP‐tagged Cacophony (Gratz et al., 2019; Li, Goel, Chen, et al., 2018), implying a sustained increase in the levels of these proteins during PHP. In this case, three of the above‐mentioned genes that have been tested for PHP in the *GluRIIA^SP16^* mutant background—*cacophony* (Frank et al., 2006), *Brp* (Böhme et al., 2019; Penney et al., 2012), and *Srpk79D* (Böhme et al., 2019)—are required for PHP, indicating a correlation between structural and functional changes after sustained glutamate receptor perturbation. Recently, Goel et al. (2019) uncovered a role for Arl8‐dependent axonal transport of synaptic material in AZ remodeling during PHP. Similar to Böhme et al. (2019), Arl8‐dependent structural plasticity was dispensable for rapid PHP expression, but required for sustained PHP expression. Given that AZ remodeling can be uncoupled from rapid PHP expression, it remains to be determined how structural changes relate to PHP, and which mechanisms regulate the abundance of these proteins. The finding of a role for axonal transport in the regulation of synaptic protein levels constitutes an interesting starting point.

### 3.2 Retrograde, transsynaptic signaling

At the *Drosophila* NMJ, PHP likely involves retrograde signaling from the postsynaptic muscle cell to the presynaptic motor neuron (Figure 3a). Several lines of evidence support a model of retrograde PHP signaling: first, genetic manipulations targeting postsynaptic receptor function—including ablation of the GluRIIA subunit (Davis & Goodman, 1998; DiAntonio et al., 1999; Petersen et al., 1997) or postsynaptic expression of RNAi transgenes targeting glutamate receptor subunits (Brusich et al., 2015; Li, Goel, Chen, et al., 2018)—induce changes in presynaptic release. One long‐standing puzzle in the field has been the identity of “the retrograde signal” that conveys information from the muscle to the nerve—or alternatively, the multiple signals that are used by the NMJ at distinct sites or developmental time points. Because PHP is quantitative and precise, these signaling pathways likely contain information about the magnitude of the postsynaptic impairment.

Transsynaptic signaling processes have been characterized that satisfy some of the requirements of a retrograde signal. *Drosophila* multiplexin is one such molecule. Multiplexin is a homolog of Collagen XV/XVIII; in the context of tumor cell lines, it is known that Collagen XVIII is cleaved to release the anti‐angiogenesis factor endostatin (Felbor et al., 2000). At the *Drosophila* NMJ, loss of multiplexin blocks both, the rapid expression and the sustained maintenance of PHP (Figure 3a; Wang, Hauswirth, Tong, Dickman, & Davis, 2014). This defect is rescued by expressing wild‐type *multiplexin* transgenes either in the muscle or the motor neuron in the mutant background (Wang et al., 2014). This suggests that multiplexin is involved in transsynaptic PHP signaling. However, based on the finding that presynaptic or postsynaptic *multiplexin* expression restores PHP, it is unknown if endogenous multiplexin is an instructive muscle‐to‐nerve retrograde PHP signal. *Drosophila* multiplexin contains Thrombospondin‐like domains (Meyer & Moussian, 2009), and in the context of vertebrate CNS synaptogenesis, Thrombospondin has been proposed to act as an extracellular signal that binds to the α2δ subunit of voltage‐gated Ca^2+^ channels (Eroglu et al., 2009). Interestingly, at the *Drosophila* NMJ, loss‐of‐function *α2δ‐3* alleles block PHP (Wang, Jones, et al., 2016), and multiplexin is required for homeostatic control of presynaptic Ca^2+^ influx (Wang et al., 2014).

A major new finding in terms of how retrograde signaling governs PHP at the *Drosophila* NMJ arose from a recent study of classical axon guidance molecules, the semaphorins and plexins, with new roles revealed for these molecules in the context of PHP (Orr, Fetter, & Davis, 2017). The semaphorin 2b (Sema2b) signaling molecule and its receptor plexin B (PlexB) act as a ligand–receptor pair required for PHP (Figure 3a; Orr et al., 2017). At the NMJ, Sema2b secreted from muscle acts upon PlexB in the neuron to induce PHP (Orr et al., 2017). This is an instructive process, as acute application of exogenous Sema2b to the NMJ induces an increase in quantal content on its own, in a way that depends upon normal PlexB activity (Orr et al., 2017). Thus, the axon guidance functions of Sema2b–PlexB may have been co‐opted in support of PHP at the NMJ. Indeed, canonical downstream activity of the cytoplasmic actin regulator Mical also mediates PHP (Orr et al., 2017).

Bone morphogenetic protein (BMP) activity is another intriguing extracellular signal. Past work has shown that BMP plays a complex set of roles at the NMJ, governing both homeostatic synaptic plasticity at the NMJ and NMJ development (Goold & Davis, 2007; Haghighi et al., 2003; McCabe et al., 2003). Recent work at the NMJ has added molecular detail, implicating a possible role at the presynapse for the BMP effector molecule Mothers against decapentaplegic (Mad). By NMJ immunostaining, phospho‐Mad (pMad) levels diminish at the presynaptic neuron when muscle GluRIIA‐containing receptors are lost (Sulkowski, Kim, & Serpe, 2014). This positive correlation between pMad protein levels at presynaptic sites and GluRIIA abundance in the muscle works in the reciprocal direction, as specific reduction of presynaptic pMad also causes a reduction of GluRIIA‐containing receptors (Sulkowski et al., 2014). Since loss of GluRIIA constitutes a well‐known homeostatic challenge that induces PHP, the BMP‐related signaling mechanisms that control neuronal pMad at synaptic sites could be relevant.

Future work should address how these transsynaptic signaling processes integrate with one another, and if other molecular pathways participate in trans‐synaptic PHP signaling. Does PHP signaling modulate postsynaptic secretion of diffusible factors, such as Sema2b (Orr et al., 2017), or does it also involve signaling via transsynaptic molecules previously implicated in PHP, such as cell‐adhesion molecules? It will be exciting to elucidate how these retrograde signaling systems encode the magnitude of glutamate receptor impairment.

### 3.3 Postsynaptic mechanisms

The majority of genes that have been implicated in PHP at the *Drosophila* NMJ were shown to regulate neurotransmitter release (Delvendahl & Müller, 2019). Comparably little is known about postsynaptic molecular mechanisms underlying PHP.

Several lines of evidence suggest a regulatory role of postsynaptic CaMKII in PHP (Figure 3a): Postsynaptic expression of a constitutively active CaMKII transgene impairs long‐term PHP expression (Haghighi et al., 2003; Li, Goel, Chen, et al., 2018). Two groups recently reported a decreased phosphorylation state of CaMKII (pCaMKII) in the muscle cell upon long‐term glutamate receptor perturbation (Goel et al., 2017; Li, Goel, Chen, et al., 2018; Newman et al., 2017; see Section 2.2. above). PHP can be induced in the absence of extracellular Ca^2+^ (Goel et al., 2017), and the decrease in pCaMKII levels after glutamate receptor inhibition occurs in the absence of extracellular Ca^2+^ (Goel et al., 2017). This indicates that decreased Ca^2+^ influx through Ca^2+^‐permeable glutamate receptors is unlikely involved in inducing PHP and reducing pCaMKII.

Further studies have demonstrated postsynaptic roles for a discrete set of molecules in PHP expression, including canonical BMP signaling components (Goold & Davis, 2007), a regulatory network of Src‐family tyrosine kinases (Spring, Brusich, & Frank, 2016), as well as the *Drosophila* homologs of proteins known to regulate cap‐dependent translation, target of rapamycin (TOR), S6 kinase (S6K), eIF43, and 4E‐BP (Kauwe et al., 2016; Penney et al., 2012). Recently, it was found that postsynaptic glutamate receptor inhibition and postsynaptic mTOR overexpression enhance release through similar presynaptic mechanisms (Figure 3a; Goel et al., 2017). However, while postsynaptic glutamate receptor impairment resulted in decreased pCaMKII levels, this was not observed after postsynaptic mTOR overexpression (Goel et al., 2017). This indicates that postsynaptic glutamate receptor impairment and mTOR signaling likely control presynaptic release through different pathways.

An electrophysiology‐based genetic screen identified a postsynaptic role for class II PI3K in PHP (Figure 3a; Hauswirth et al., 2018). The results of this study suggest that postsynaptic class II PI3K regulates endosomal PI3P levels, which in turn recruit the small GTPase Rab11 to recycling endosomes. Thus, postsynaptic vesicle trafficking likely participates in PHP. Another recent genetic screen implicated *insomniac*, a gene encoding an alleged Cullin‐3 ubiquitin ligase complex adaptor, in PHP (Kikuma et al., 2019). Postsynaptic *insomniac* was found to be required for rapid and chronic PHP expression. The study also provided evidence for rapid and local monoubiquitination at postsynaptic densities during PHP, and links between *insomniac* and postsynaptic vesicle trafficking targeting *multiplexin*, (see Section 3.2.; Wang et al., 2014). In summary, while there has been a significant progress in uncovering molecular substrates of postsynaptic PHP signaling, several major questions remain: Which postsynaptic parameter is sensed during PHP, and how do the molecular pathways identified so far intersect with each other, as well as transsynaptic signaling?

## 4 OPEN QUESTIONS AND OUTLOOK

Despite considerable progress in identifying the physiological and molecular underpinnings of homeostatic regulation of neurotransmitter release at the *Drosophila* NMJ, several outstanding questions remain unanswered. We consider the following three major open questions as especially interesting:

1. *PHP induction and maintenance—*It is currently completely unknown which parameters are “sensed” during PHP. What are the biological substrates of PHP induction? How do these mechanisms relate to the rapid and sustained expression of PHP? A recent study, which provides evidence for a functional separation between the induction and maintenance phases of PHP (James, Zwiefelhofer, & Frank, 2019), constitutes an interesting starting point for future work.
2. *Structural plasticity and proteostasis—*Genetic screens have identified a number of genes that are required for PHP at the *Drosophila* NMJ. Nevertheless, it is largely unclear how the corresponding proteins are regulated during PHP. Recent data suggests modulation of the abundance of several presynaptic proteins during PHP (Böhme et al., 2019), and an involvement of axonal transport (Böhme et al., 2019; Goel et al., 2019). However, many of these proteins are not required for PHP expression on rapid time scales. Another related open question concerns the role of synaptic proteostasis during PHP. While impairing the presynaptic UPS blocks PHP (Wentzel et al., 2018), it remains to be determined how the UPS participates in PHP signaling.
3. *Evolutionary conservation and disease relevance—*There is evidence for PHP‐like phenomena at various synapses in different species, but it is unknown how much is shared mechanistically. For chronic challenges to synapse function, there are some indications for conserved homeostatic mechanisms. These responses include retrograde signaling mediated by TOR (Henry et al., 2012), a requirement for presynaptic Ca^2+^‐channel activity to effect potentiation (Jakawich et al., 2010), as well as changes in presynaptic Ca^2+^ influx that offset various perturbations (Zhao et al., 2011) or vesicle pool size (Jeans et al., 2017; Kim & Ryan, 2010). Less is known about the conservation of rapid forms of homeostatic plasticity. A rapid form of PHP akin that at the *Drosophila* NMJ has been discovered at mammalian NMJs (Wang et al., 2018; Wang, Pinter, et al., 2016). A similar mode of rapid PHP has not been described for CNS synapses. Yet, shared mechanisms between the *Drosophila* NMJ and mammalian CNS synapses could be at play. PHP at the *Drosophila* NMJ requires retrograde Sema2b–PlexB signaling to the actin regulator Mical (Orr et al., 2017) or control of small GTPase activity by the Rho‐type guanine exchange factor Ephexin (Frank, Pielage, & Davis, 2009). These signals are reminiscent of events previously described at mammalian synapses to regulate vesicle pool size—and possibly, homeostatic plasticity. For example, inhibition of the cytoskeletal regulator myosin light chain kinase (MLCK) increases RRP size at the mouse calyx of Held (Srinivasan, Kim, & Gersdorff, 2008). Related work suggests that tonic inhibition of MLCK by Rho‐associated kinase (ROCK) underlies a mechanism of RRP maintenance (González‐Forero et al., 2012). It will be exciting to test if these molecular mechanisms also participate in PHP. Intriguingly, there is evidence for presynaptic potentiation of release after sustained glutamate receptor inhibition at the calyx of Held (Yang et al., 2011). Thus, this synapse provides an intriguing entry point to test if MLCK signaling participates in PHP.

Given that most genes that are required for PHP at the *Drosophila* NMJ have been implicated in various neural disorders (Wondolowski & Dickman, 2013)—and given emerging links between PHP signaling and factors involved in critical everyday functions, such as sleep (Kikuma et al., 2019)—it will be exciting to explore potential roles of homeostatic synaptic plasticity in the physiology and pathophysiology of neural function.

## CONFLICT OF INTEREST

The authors declare no conflicts of interest.
